# Supplementary material for: Design, Synthesis, and Evaluation of Near-Infrared Fluorescent Molecules Based on 4H-1-Benzopyran Core
Source: Molecules. 2021 Nov 19;26(22):6986. doi: 10.3390/molecules26226986 (PMC8620761; doi:10.3390/molecules26226986)
Supplement: Supplementary file 1 [file molecules-26-06986-s001.zip › molecules-1455680-supplementary.pdf]

Supporting Information

Design, Synthesis, and Evaluation of Near-infrared Fluorescent Molecules Based on 4H-1-Benzopyran Core

Shuting Wang [1,2,†], Shulei Zhu [1,†], Yawen Tanzeng [3], Yuexing Zhang [4], Chuchu Li [1], Mingliang Ma [1,2,*], Wei Lu [1]

[1]Shanghai Engineering Research Center of Molecular Therapeutics and New Drug Development, School of Chemistry and Molecular Engineering, East China Normal University, Shanghai 200062, China.

[2]School of Ecological and Environmental Sciences, East China Normal University, Shanghai 200241, China

[3]Key Laboratory of Brain Functional Genomics-Ministry of Education, School of Life Science, East China Normal University, Shanghai 200062, China.

[4]Collaborative Innovation Center for Advanced Organic Chemical Materials Co-constructed by the Province and Ministry, Ministry of Education Key Laboratory for the Synthesis and Application of Organic Functional Molecules, College of Chemistry and Chemical Engineering, Hubei University, Wuhan 430062, PR China

*Corresponding authors.

†These authors contribute to the paper equally.

Content

1. ^{1}H NMR and ^{13}C NMR spectra for M series 2

2. HR-MS Data 8

3. HPLC spectra assessment of purity 10

4. UV absorption spectrum and fluorescence spectrum 11

5. Supporting table data 12

6. Optimized geometry and xyz coordinates…..……………………………..... 14

1. ^{1}H NMR and ^{13}C NMR spectra for M series

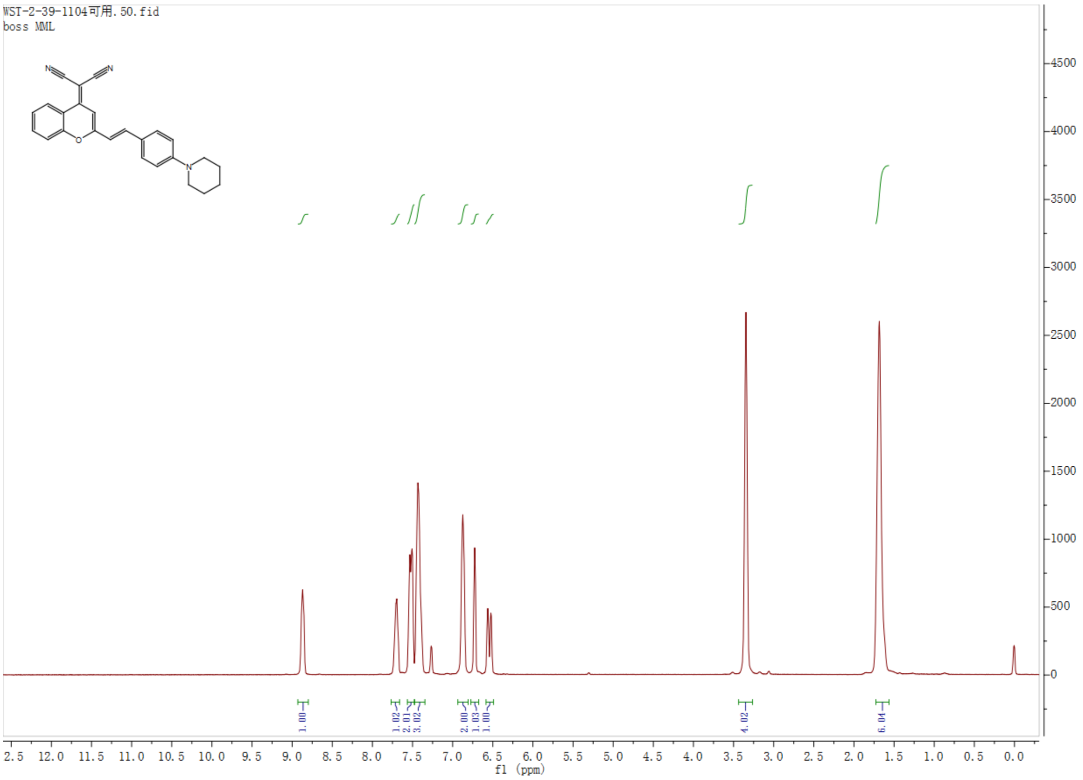

^{1}H NMR (400 MHz, Chloroform-d) δ 8.87 (s, 1H), 7.71 (s, 1H), 7.52 (d, J = 10.9 Hz, 2H), 7.43 (s, 3H), 6.87 (s, 2H), 6.73 (s, 1H), 6.54 (d, J = 15.9 Hz, 1H), 3.35 (s, 4H), 1.68 (s,6H).

Figure S1. ^{1}H NMR (400 MHz) of **M-1** in $CDCl_3$

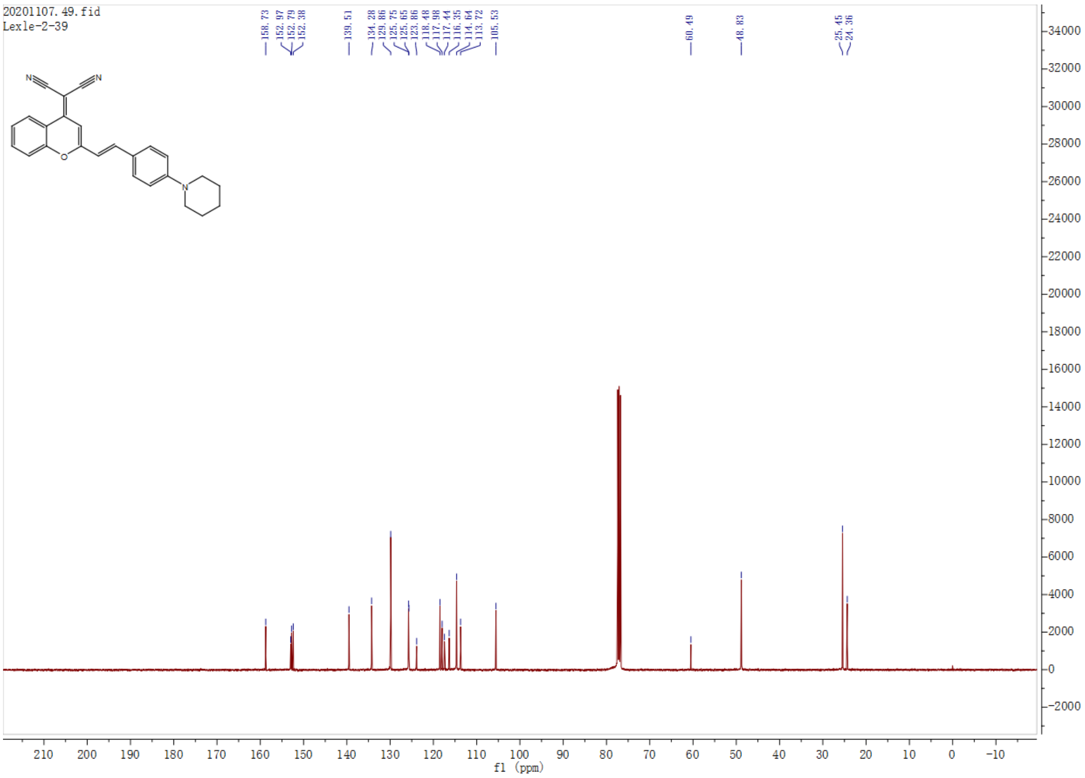

^{13}C NMR (101 MHz, Chloroform-d) δ 158.7, 153.0, 152.8, 152.4, 139.5, 134.3, 129.9, 125.7, 123.9, 118.5, 118.0, 117.4, 116.4, 114.6, 113.7, 105.5

Figure S2. ^{13}C NMR (400 MHz) of **M-1** in $CDCl_3$

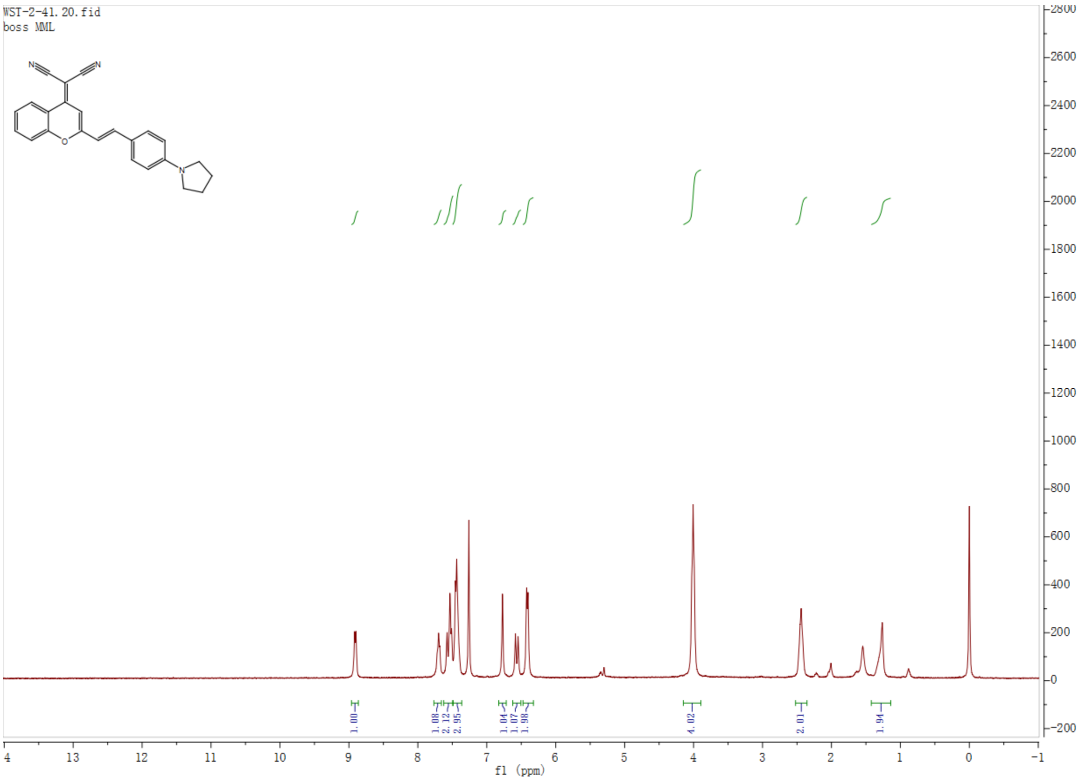

^{1}H NMR (400 MHz, Chloroform-d) δ 8.91 (d, J = 8.1 Hz, 1H), 7.70 (t, J = 7.7 Hz, 1H), 7.54 (t, J = 13.4 Hz, 2H), 7.44 (t, J = 7.8 Hz, 3H), 6.77 (s, 1H), 6.56 (d, J = 15.6 Hz, 1H), 6.41 (d, J = 8.2 Hz, 2H), 4.01 (t, J = 7.3 Hz, 4H), 2.58 – 2.33 (m, 2H), 1.26 (s, 2H).

Figure S3. ^{1}H NMR (400 MHz) of **M-2** in $CDCl_3$

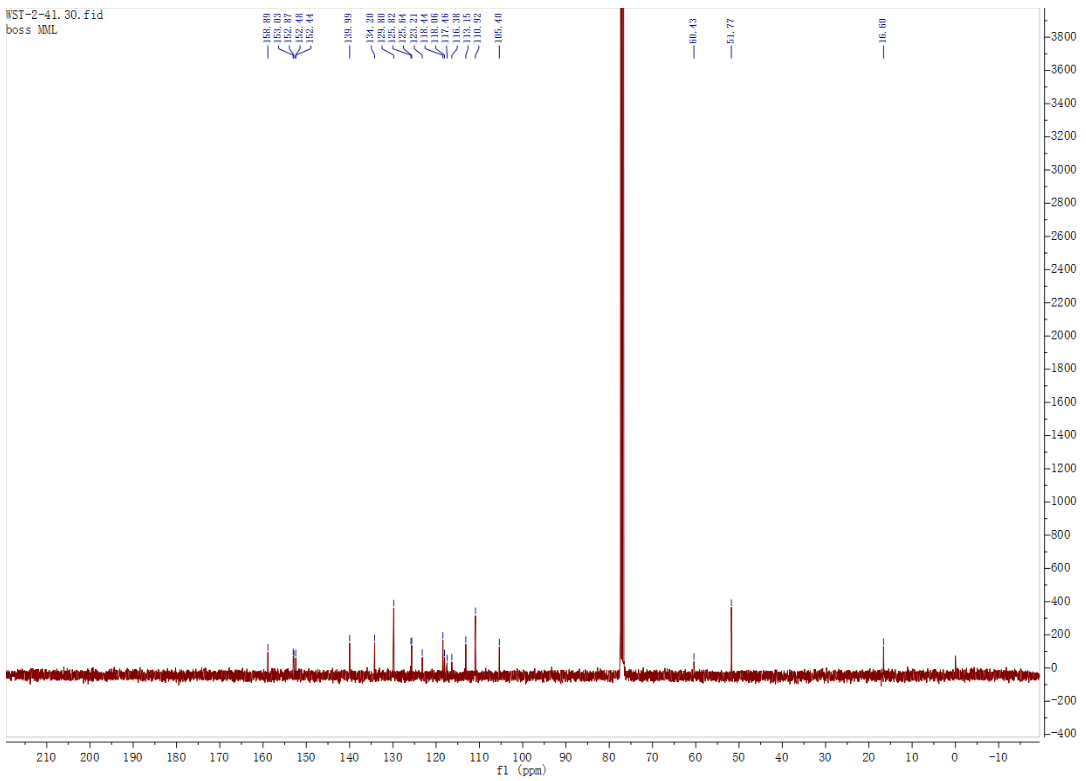

^{13}C NMR (101 MHz, Chloroform-d) δ 158.9, 153.0, 152.9, 152.4, 140.0, 134.2, 129.8, 125.8, 125.6, 123.2, 118.4, 118.1, 117.5, 116.4, 113.2, 110.9, 105.4, 60.4, 51.8, 16.6.

Figure S4. ^{13}C NMR (400 MHz) of **M-2** in $CDCl_3$

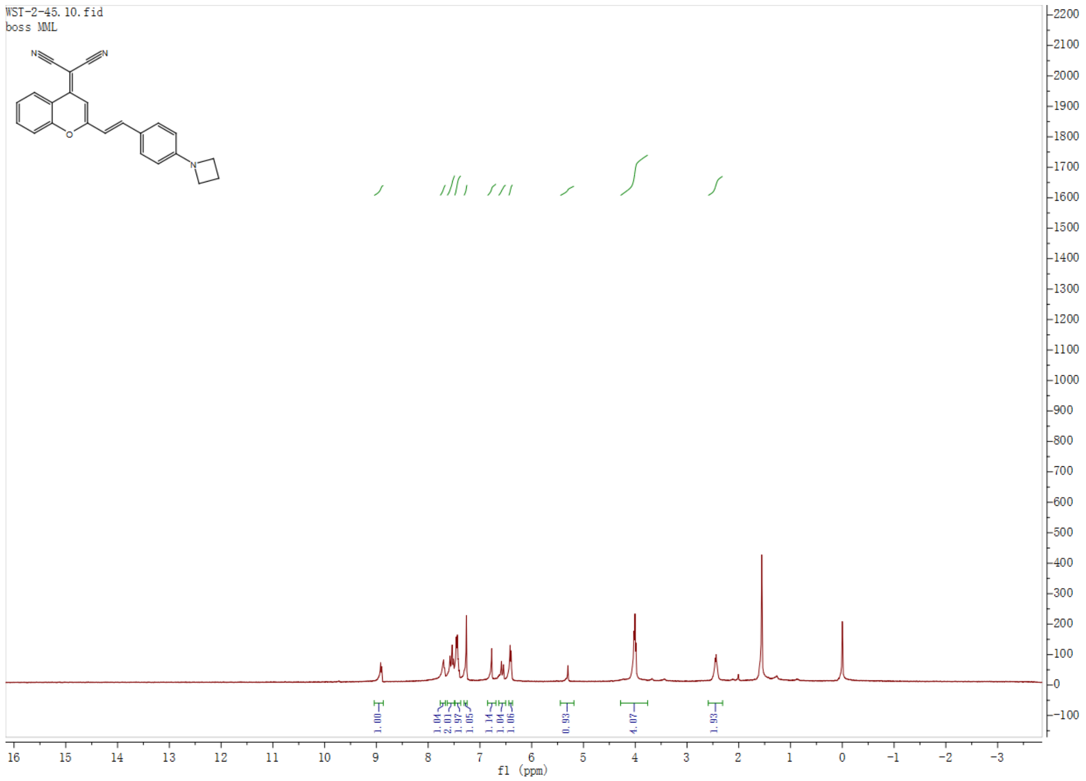

^{1}H NMR (400 MHz, Chloroform-d) δ 8.91 (d, J = 8.6 Hz, 1H), 7.85 – 7.64 (m, 1H), 7.63 – 7.50 (m, 2H), 7.45 (d, J = 8.2 Hz, 2H), 7.26 (d, J = 1.7 Hz, 1H), 6.77 (s, 1H), 6.57 (d, J = 15.8 Hz, 1H), 6.41 (d, J = 8.3 Hz, 1H), 5.30 (s, 1H), 4.26 – 3.74 (m, 4H), 2.59 – 2.33 (m, 2H).

Figure S5. ^{1}H NMR (400 MHz) of **M-3** in $CDCl_3$

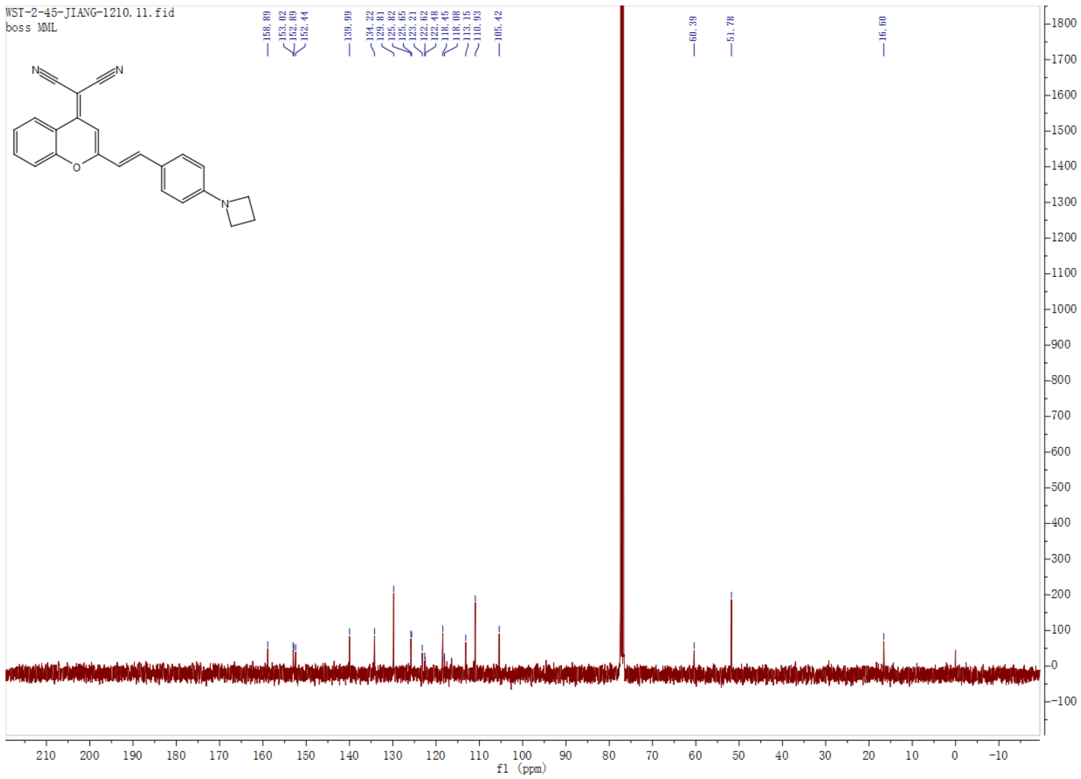

^{13}C NMR (400 MHz, Chloroform-d) δ 140.0, 134.2, 129.8, 125.8, 125.7, 123.2, 122.6, 118.5, 113.2, 110.9, 105.4, 60.4, 51.8, 16.6.

Figure S6. ^{13}C NMR (400 MHz) of **M-3** in $CDCl_3$

2. HR-MS Data for M series

Elemental Composition Report **Page 1**

Single Mass Analysis
Tolerance = 500.0 mDa / DBE: min = -1.5, max = 50.0
Element prediction: Off

Monoisotopic Mass, Even Electron Ions
3 formula(e) evaluated with 1 results within limits (up to 50 closest results for each mass)
Elements Used:
C: 25-25 H: 11-54 N: 3-3 O: 1-1 Na: 0-1 I: 0-1

Default file
WST-2-39 41 (0.832)
1: TOF MS ES+
3.62e+002

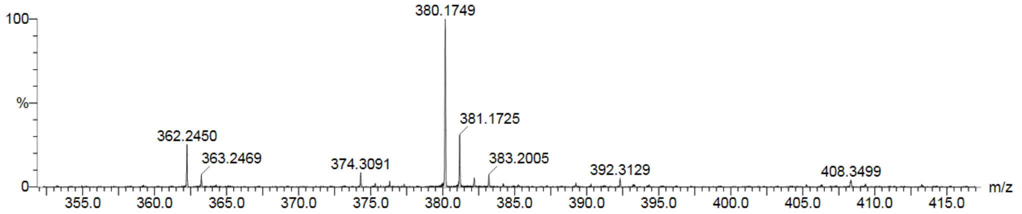

Minimum:				-1.5	
Maximum:		500.0	10.0	50.0	
Mass	Calc. Mass	mDa	PPM	DBE	Formula
380.1749	380.1763	-1.4	-3.7	16.5	C25 H22 N3 O

Figure S7. The HR-MS spectrum of **M-1**

Elemental Composition Report **Page 1**

Single Mass Analysis
Tolerance = 500.0 mDa / DBE: min = -1.5, max = 50.0
Element prediction: Off

Monoisotopic Mass, Even Electron Ions
3 formula(e) evaluated with 1 results within limits (up to 50 closest results for each mass)
Elements Used:
C: 24-24 H: 11-54 N: 3-3 O: 1-1 Na: 0-1 I: 0-1

WST-2-41 26 (0.537) Cm (25:62)
1: TOF MS ES+
1.30e+003

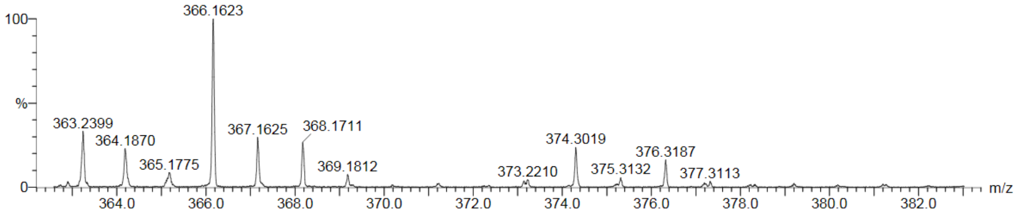

Minimum:				-1.5	
Maximum:		500.0	10.0	50.0	
Mass	Calc. Mass	mDa	PPM	DBE	Formula
366.1623	366.1606	1.7	4.6	16.5	C24 H20 N3 O

Figure S8. The HR-MS spectrum of **M-2**

Elemental Composition Report　　Page 1

Single Mass Analysis
Tolerance = 500.0 mDa / DBE: min = -1.5, max = 50.0
Element prediction: Off

Monoisotopic Mass, Even Electron Ions
3 formula(e) evaluated with 1 results within limits (up to 50 closest results for each mass)
Elements Used:
C: 23-25 H: 11-54 N: 3-3 O: 1-1 Na: 0-1 I: 0-1

WST-2-45A 37 (0.744)
1: TOF MS ES+

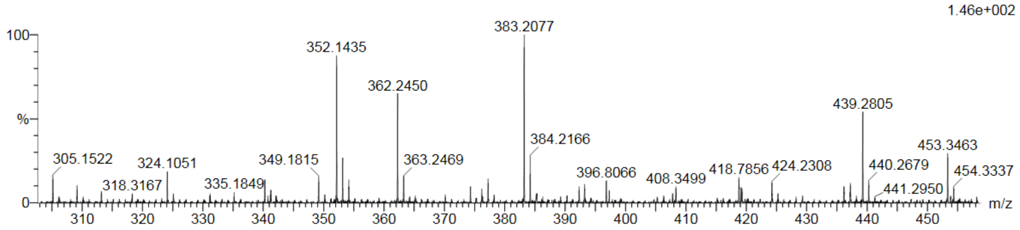

Minimum:				-1.5	
Maximum:		500.0	10.0	50.0	
Mass	Calc. Mass	mDa	PPM	DBE	Formula
352.1435	352.1450	-1.5	-4.3	16.5	C23 H18 N3 O

Figure S9. The HR-MS spectrum of **M-3**

3. HPLC spectra assessment of purity for M series

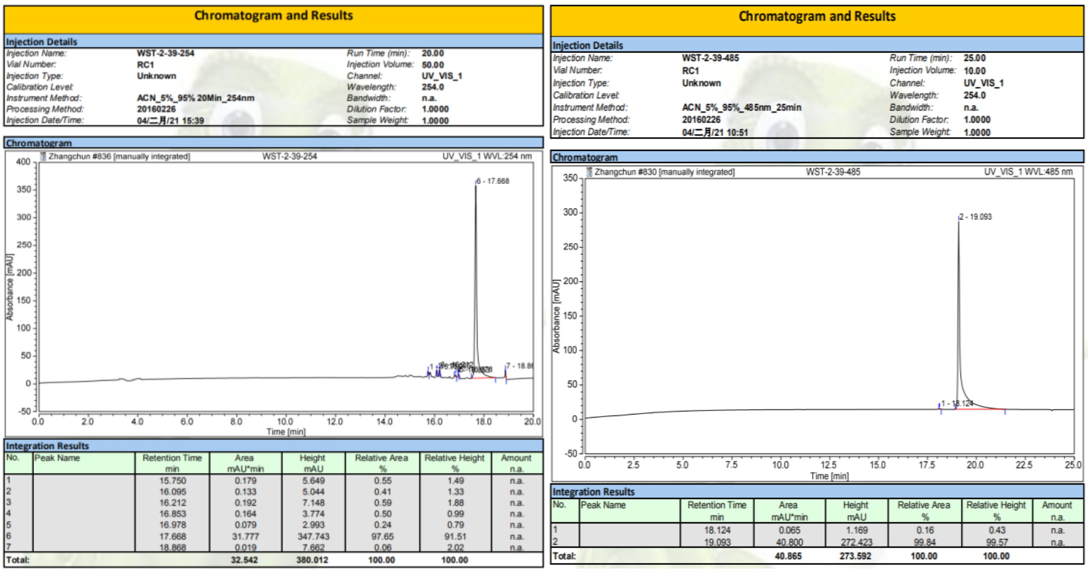

Integration Results (254 nm)

No.	Peak Name	Retention Time min	Area mAU*min	Height mAU	Relative Area %	Relative Height %	Amount n.a.
1		15.750	0.179	5.649	0.55	1.49	n.a.
2		16.095	0.133	5.044	0.41	1.33	n.a.
3		16.212	0.192	7.148	0.59	1.88	n.a.
4		16.853	0.164	3.774	0.50	0.99	n.a.
5		16.978	0.079	2.993	0.24	0.79	n.a.
6		17.668	31.777	347.743	97.65	91.51	n.a.
7		18.868	0.019	7.662	0.06	2.02	n.a.
Total:			32.542	380.012	100.00	100.00	

Integration Results (485 nm)

No.	Peak Name	Retention Time min	Area mAU*min	Height mAU	Relative Area %	Relative Height %	Amount n.a.
1		18.124	0.065	1.169	0.16	0.43	n.a.
2		19.093	40.800	272.423	99.84	99.57	n.a.
Total:			40.865	273.592	100.00	100.00	

Figure S10. The HPLC spectrum of **M-1** under 254nm and 485nm

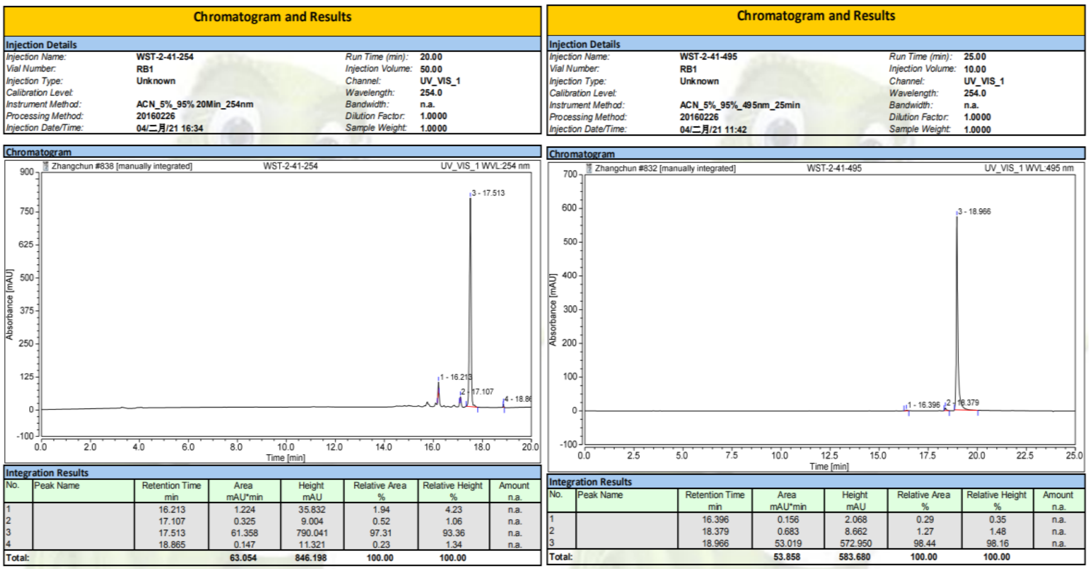

Integration Results (254 nm)

No.	Peak Name	Retention Time min	Area mAU*min	Height mAU	Relative Area %	Relative Height %	Amount n.a.
1		16.213	1.224	35.832	1.94	4.23	n.a.
2		17.107	0.325	9.004	0.52	1.06	n.a.
3		17.513	61.358	790.041	97.31	93.36	n.a.
4		18.865	0.147	11.321	0.23	1.34	n.a.
Total:			63.054	846.198	100.00	100.00	

Integration Results (495 nm)

No.	Peak Name	Retention Time min	Area mAU*min	Height mAU	Relative Area %	Relative Height %	Amount n.a.
1		16.396	0.156	2.068	0.29	0.35	n.a.
2		18.379	0.683	8.662	1.27	1.48	n.a.
3		18.966	53.019	572.950	98.44	98.16	n.a.
Total:			53.858	583.680	100.00	100.00	

Figure S11. The HPLC spectrum of **M-2** under 254nm and 495nm

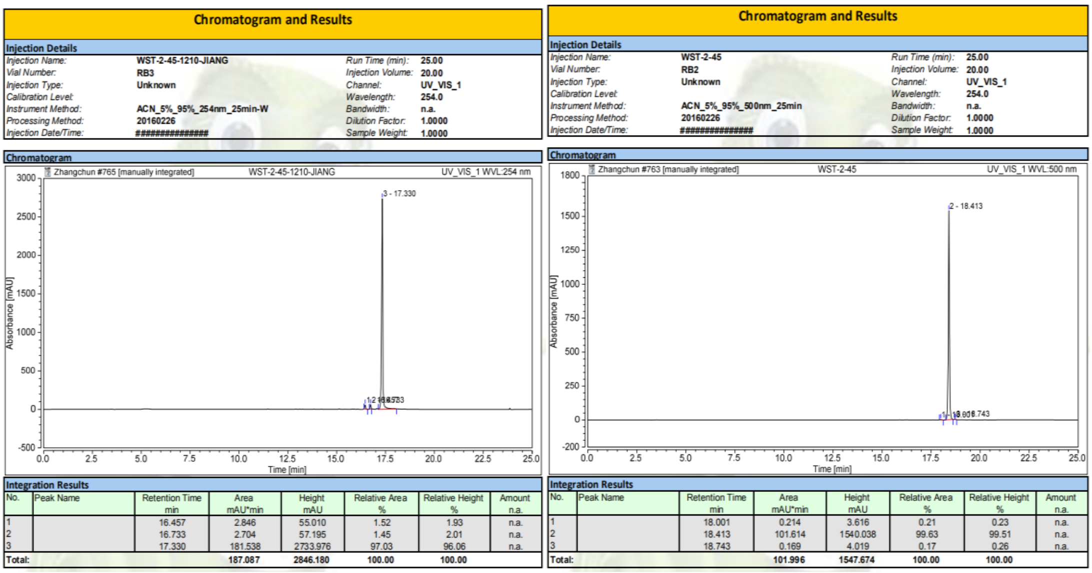

Figure S12. The HPLC spectrum of **M-3** under 254nm and 500nm

4. UV absorption spectrum and fluorescence spectrum for M series

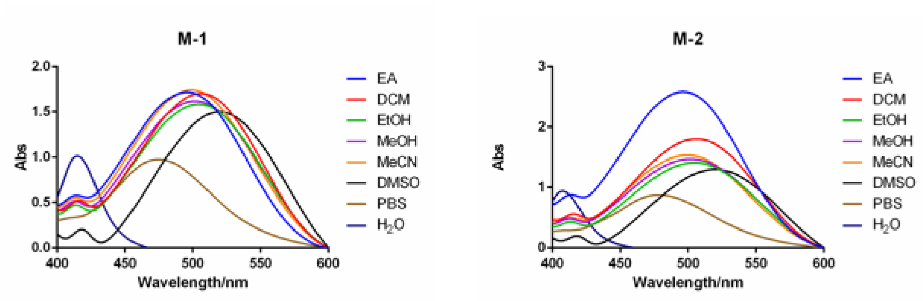

Figure S13. UV absorption spectrum of 50uM **M-1** and **M-2** in different solvent

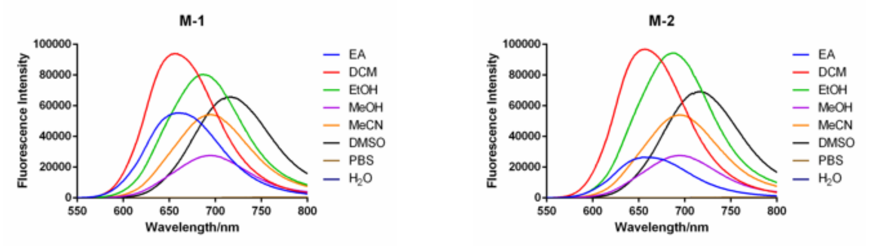

Figure S14. Fluorescence emission spectrum of 50uM **M-1** and **M-2** in different solvent

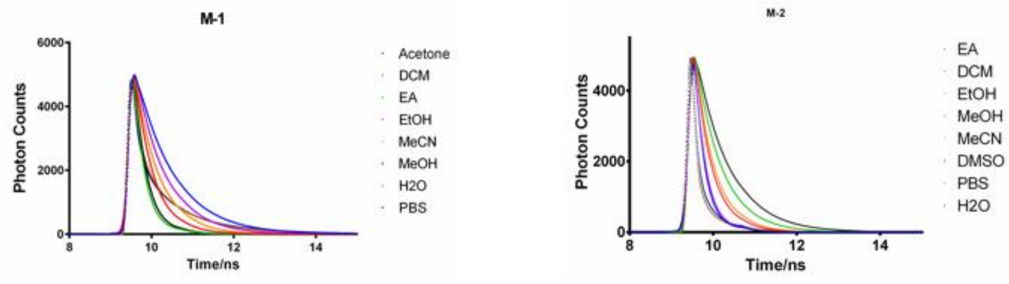

Figure S15. The fluorescence decays of **M-1** and **M-2** in different solvent

5. Supporting table data

Table S1. M-1 spectrum data

a

Solvent	λ_{b}^{a}(nm)	λ_{c}^{a}(nm)	△λ(nm)	Q_{Ein}	AF
EA	495	660	165	0.0401	0.5037
DCM	506	655	149	0.0512	0.2343
EtOH	504	687	183	0.0437	0.2661
MeOH	501	695	194	0.0141	0.3254
MeCN	498	693	195	0.0306	0.4139
DMSO	522	718	196	0.0412	0.4600
H_2O	474	781	307	N	N
PBS	422	788	366	N	N

maximum
[b] absorption
[c] emission

Table S2. M-2 spectrum data

Solvent	λ_{b}^{a}(nm)	λ_{c}^{a}(nm)	△λ(nm)	Q_{Ein}	AF
EA	496	656	160	0.0390	0.3890
DCM	506	655	149	0.0480	0.2999
EtOH	504	689	185	0.0364	0.4157
MeOH	502	695	193	0.0140	0.5711
MeCN	499	693	194	0.0267	0.5376
DMSO	519	711	192	0.0387	0.4623
H_2O	478	781	303	N	N
PBS	469	794	325	N	N

maximum
[b] absorption
[c] emission

Table S3. M-1 average lifetime in different solvents

M-1	Average Lifetime（ns）	Chisqr
Acetone	0.82612	1.08153
DCM	0.42287	1.09183

EA	0.24170	0.78509
EtOH	0.66948	1.02223
H_2O	0.93379	1.12571
MeCN	0.51774	1.03802
MeOH	0.27311	0.71691
PBS	0.98113	1.10881

Table S4. M-2 average lifetime in different solvents

**M-2**	Average Lifetime（ns）	Chisqr
Acetone	0.73869	1.045
DCM	0.41942	1.08201
EA	0.23657	0.97067
EtOH	0.57178	1.06442
H_2O	0.24525	1.06974
MeCN	0.42679	0.89964
MeOH	0.2488	0.81336
PBS	0.37446	1.03882

Table S5. M-3 average lifetime in different solvents

**M-3**	Average Lifetime（ns）	Chisqr
DCM	0.40738	0.96977
EA	0.29433	0.91535
EtOH	0.58272	1.00478
MeOH	0.2565	0.88928
MeCN	0.43155	0.96132
H_2O	0.45366	1.17758
PBS	0.39437	1.03882

Table S6. M-1~3 average lifetime in BSA

in BSA	Average Lifetime（ns）	Chisqr
**M-1**	2.18788	1.03131
**M-2**	2.05296	1.01324
**M-3**	2.33991	0.98738

Table S7. M-3 average lifetime in PEG

**M-3**	Average Lifetime（ns）	Chisqr
PEG0	0.59973	1.06925
PEG10	0.65729	1.03483
PEG20	0.7109	1.0342
PEG30	0.76625	1.14293
PEG40	0.79901	1.10521
PEG50	0.87558	1.08563

PEG60	0.95043	1.11962
PEG70	1.13675	1.0369
PEG80	1.16695	1.07749
PEG90	1.30254	1.10788
PEG100	1.40621	1.03194

6. Optimized geometry and xyz coordinates

Optimized geometry of **M-1**, **M-2**, **M-3** were zipped as " M-1-3_ES_GS_MOL-SI.zip" which would be uploaded independently.

Table S8. The xyz coordinates of the optimized geometry at B3LYP/6-31g(d,p)-IEFPCM(water) level of all molecules in both ground (GS) and first excited states (ES).

**M-1** GS				**M-1** ES			
C	-5.54646391	-2.61796792	0.04185830	C	-5.61839545	-2.56849203	-0.00106831
C	-4.45223931	-3.49423821	0.03150290	C	-4.55603701	-3.47612169	-0.00067392
C	-3.16234353	-2.98636129	0.01291842	C	-3.24640837	-2.99682792	-0.00023220
C	-2.97246922	-1.60277774	0.00488969	C	-3.01150046	-1.62515879	-0.00012643
C	-4.04903972	-0.68723982	0.01526577	C	-4.06460069	-0.67006272	-0.00037416
C	-5.34696086	-1.24577988	0.03388245	C	-5.37752228	-1.19810460	-0.00093793
O	-1.66475877	-1.20166699	-0.01385671	O	-1.68440657	-1.26431559	0.00020380
C	-1.35524748	0.11827234	-0.02402662	C	-1.34953494	0.06757218	-0.00011761
C	-2.35406052	1.06824492	-0.01485703	C	-2.32889686	1.03559255	-0.00029272
C	-3.73343825	0.74550506	0.00580026	C	-3.71837861	0.74435130	-0.00018351
C	-4.67803637	1.79812945	0.01582591	C	-4.64429496	1.84573595	0.00008845
C	0.04167560	0.42531372	-0.04350770	C	0.05172659	0.34173321	-0.00010213
C	1.02973903	-0.51621385	-0.04967831	C	1.03803212	-0.61430535	0.00027747
C	2.45298287	-0.29815985	-0.06745481	C	2.45909327	-0.36590853	0.00028530
C	3.31993679	-1.41774069	-0.07057653	C	3.35226956	-1.47436715	0.00104294
C	4.69472961	-1.28253030	-0.08563717	C	4.71933067	-1.31155609	0.00110648
C	5.28368444	0.00762736	-0.09914486	C	5.28079161	-0.00148580	0.00038985
C	4.42609929	1.14173263	-0.09692566	C	4.40133561	1.12138454	-0.00037978
C	3.05578426	0.98509222	-0.08135126	C	3.03779352	0.93707678	-0.00043538
N	6.62870711	0.15876890	-0.13057940	N	6.61445298	0.17288527	0.00045199
C	7.47251056	1.34062651	0.08789605	C	7.46022370	1.37311124	-0.00074696
C	8.69356235	0.38944377	0.22667810	C	8.70639098	0.44683250	-0.00070424
C	7.71098899	-0.80726175	0.09539710	C	7.74085010	-0.76928249	0.00053718
C	-4.23141954	3.14905850	0.00458440	C	-4.16125392	3.17662813	-0.00054455
N	-3.87399638	4.26098212	-0.00441235	N	-3.76520629	4.27953013	-0.00110016
C	-6.09076896	1.68293517	0.03768699	C	-6.05381445	1.77414575	0.00138275
N	-7.25948274	1.66850602	0.05554063	N	-7.22671074	1.79019293	0.00267815
H	-6.55745419	-3.01073398	0.05617283	H	-6.64249980	-2.92825365	-0.00150098
H	-4.60902314	-4.56779849	0.03783045	H	-4.74067275	-4.54532458	-0.00075142
H	-2.29091251	-3.63132954	0.00426481	H	-2.39395555	-3.66757380	0.00000294
H	-6.21072551	-0.59792327	0.04209250	H	-6.22200116	-0.52450465	-0.00131646

H	-2.04500197	2.10561400	-0.02339613	H	-1.99717196	2.06731393	-0.00044332
H	0.27281576	1.48517293	-0.05265065	H	0.30265137	1.39767189	-0.00038359
H	0.72089296	-1.55938460	-0.03977912	H	0.73482987	-1.65704630	0.00062785
H	2.88788834	-2.41507389	-0.05909581	H	2.93778474	-2.47801734	0.00159280
H	5.32929232	-2.16243468	-0.08882166	H	5.37385063	-2.17609171	0.00168907
H	4.85813783	2.13693595	-0.10901130	H	4.81572884	2.12361210	-0.00094718
H	2.43392963	1.87491835	-0.07836023	H	2.39822887	1.81267949	-0.00107749
H	7.21384321	1.89811562	0.99694275	H	7.32557994	1.99580367	0.89105627
H	7.49601307	2.03555122	-0.75898988	H	7.32472881	1.99458089	-0.89327315
H	9.41295103	0.46659655	-0.58928511	H	9.32769555	0.52760737	-0.89224957
H	9.21713503	0.45093607	1.18081981	H	9.32855732	0.52879441	0.89013029
H	7.57980262	-1.40149658	1.00857831	H	7.77091424	-1.40439419	0.89324287
H	7.88665298	-1.48639113	-0.74645863	H	7.77005322	-1.40553691	-0.89138259
**M-2** GS				**M-2** ES			
C	-5.93194509	-2.59737370	-0.02967378	C	-6.00574293	-2.54190564	-0.02020854
C	-4.84366008	-3.48099020	-0.03714264	C	-4.95151251	-3.45885713	-0.03061751
C	-3.55019682	-2.98187902	-0.03090375	C	-3.63768796	-2.99130008	-0.02855468
C	-3.35090007	-1.59968515	-0.01698736	C	-3.39047715	-1.62181219	-0.01613100
C	-4.42128907	-0.67690848	-0.00831686	C	-4.43507225	-0.65754407	-0.00510011
C	-5.72306571	-1.22660311	-0.01561207	C	-5.75255877	-1.17376280	-0.00782288
O	-2.04031487	-1.20744741	-0.01265843	O	-2.06026852	-1.27286613	-0.01532292
C	-1.72177064	0.11038553	-0.00132582	C	-1.71359981	0.05598816	-0.00435375
C	-2.71434815	1.06705500	0.00759425	C	-2.68455321	1.03267066	0.00642921
C	-4.09588895	0.75362165	0.00659770	C	-4.07626970	0.75391006	0.00748621
C	-5.03362310	1.81249498	0.01993601	C	-4.99242013	1.86343324	0.02077806
C	-0.32286931	0.40806997	0.00115549	C	-0.31029122	0.31821408	-0.00396540
C	0.65940079	-0.53967291	-0.00473677	C	0.66873513	-0.64512829	-0.01353585
C	2.08358002	-0.32935464	-0.00235405	C	2.09140874	-0.40487562	-0.01127894
C	2.94705174	-1.45022872	-0.00803856	C	2.98093350	-1.51418758	-0.02190772
C	4.32258204	-1.32207440	-0.00655683	C	4.34914810	-1.35742588	-0.01794317
C	4.93008802	-0.03660217	0.00098615	C	4.92910539	-0.05294160	-0.00465160
C	4.06718384	1.09782207	0.00756383	C	4.04460220	1.06879444	0.00402115
C	2.69600227	0.94817853	0.00576312	C	2.67967079	0.89200089	0.00189063
N	6.28184360	0.10781918	0.00272340	N	6.27374232	0.11557034	0.00015553
C	6.97035305	1.40364170	-0.08652182	C	6.94271518	1.42530624	-0.08226097
C	8.43235770	1.02022731	-0.35094598	C	8.41299072	1.07220249	-0.33625535
C	8.57572907	-0.33877521	0.35075243	C	8.57824166	-0.28445314	0.36396566
C	7.22420604	-1.01666326	0.08987355	C	7.24429916	-0.98886929	0.09011463
C	-4.57781944	3.16030766	0.03246454	C	-4.49755702	3.18988977	0.03023831
N	-4.21269943	4.26972929	0.04266533	N	-4.09161831	4.28916164	0.03798575
C	-6.44719048	1.70658818	0.02454937	C	-6.40248103	1.80423533	0.02699277
N	-7.61611222	1.69968248	0.02952628	N	-7.57518211	1.83026612	0.03313158
H	-6.94568434	-2.98323072	-0.03499348	H	-7.03303600	-2.89244195	-0.02182562
H	-5.00778610	-4.55341889	-0.04802354	H	-5.14564737	-4.52633498	-0.04031265

H	-2.68312652	-3.63272806	-0.03673418	H	-2.79129054	-3.66961950	-0.03648555
H	-6.58249597	-0.57297324	-0.01047510	H	-6.59101154	-0.49270834	-0.00016595
H	-2.39823074	2.10228922	0.01711542	H	-2.34377182	2.06141346	0.01496319
H	-0.08440779	1.46632713	0.00873595	H	-0.05056756	1.37200053	0.00531499
H	0.34444523	-1.58106632	-0.01176541	H	0.35888140	-1.68586924	-0.02285131
H	2.51287127	-2.44673762	-0.01789918	H	2.56504011	-2.51717936	-0.03702811
H	4.93881180	-2.21267621	-0.02259980	H	4.98547786	-2.23315272	-0.03807544
H	4.48938104	2.09524562	0.02540751	H	4.44804260	2.07334849	0.02617839
H	2.08140506	1.84308672	0.01499669	H	2.04772449	1.77307057	0.01490583
H	6.86378887	1.96472736	0.85271303	H	6.81302495	1.96778022	0.86429538
H	6.54792011	2.01477901	-0.89073952	H	6.50382372	2.02922312	-0.88154531
H	8.59852682	0.90906999	-1.42777985	H	8.59086428	0.97062223	-1.41162032
H	9.12907374	1.77441705	0.02203300	H	9.08674358	1.84046804	0.04833578
H	8.71817544	-0.19551195	1.42718640	H	8.71045395	-0.14410544	1.44154810
H	9.41269391	-0.93211379	-0.02443773	H	9.42609040	-0.86182512	-0.01001727
H	7.23328815	-1.58793938	-0.84929721	H	7.26489665	-1.54231023	-0.85865429
H	6.93883916	-1.70207283	0.89469838	H	6.95805491	-1.68592970	0.88273859
**M-3** GS				**M-3** ES			
C	-6.17428366	-2.63836586	0.22886172	C	-6.30536425	-2.56807461	0.18372647
C	-5.07614688	-3.50843966	0.17941413	C	-5.24483665	-3.47754108	0.16206091
C	-3.79230425	-2.99371566	0.08535955	C	-3.93585541	-3.00097251	0.09666900
C	-3.61233087	-1.60947585	0.04158300	C	-3.69964473	-1.63015286	0.05420663
C	-4.69303027	-0.69999455	0.09024782	C	-4.75092320	-0.67323246	0.07538287
C	-5.98463712	-1.26549701	0.18504290	C	-6.06323529	-1.19854421	0.14122147
O	-2.30997289	-1.20142565	-0.05176237	O	-2.37334777	-1.27211857	-0.00950610
C	-2.01047295	0.11971144	-0.10276020	C	-2.03825880	0.05872344	-0.06151334
C	-3.01331936	1.06402166	-0.06041781	C	-3.01525614	1.02814226	-0.04315035
C	-4.38788550	0.73399076	0.03844312	C	-4.40372662	0.74024864	0.02752949
C	-5.33709747	1.78136091	0.07957257	C	-5.32747941	1.84316152	0.04492501
C	-0.61789478	0.43415497	-0.19868135	C	-0.63765592	0.33029560	-0.13522605
C	0.37484798	-0.50110632	-0.23477599	C	0.34830225	-0.62388496	-0.14898639
C	1.79334032	-0.27135355	-0.32880878	C	1.76784599	-0.36913860	-0.22733020
C	2.67539170	-1.37424920	-0.34326844	C	2.67409016	-1.46139668	-0.19461261
C	4.04752319	-1.22475021	-0.42587780	C	4.03932550	-1.28810701	-0.26673847
C	4.64484225	0.06306104	-0.50821561	C	4.61073059	0.01655495	-0.37488784
C	3.75474881	1.17729474	-0.49205835	C	3.70184308	1.11886359	-0.40990832
C	2.38832702	1.01074146	-0.40728138	C	2.33995286	0.92756216	-0.34096199
N	6.00461340	0.23189447	-0.63746509	N	5.96406488	0.20222131	-0.45144623
C	6.93701631	-0.90004128	-0.70111420	C	6.91950276	-0.87781913	-0.72577197
C	7.57977526	-1.17431295	0.66663973	C	7.86361456	-1.10398529	0.46560705
C	8.28404668	0.09086560	1.18279973	C	8.58505069	0.20026425	0.82856514
C	7.34611202	1.30743976	1.14255073	C	7.58055850	1.34175456	1.02649313
C	6.69766515	1.46707339	-0.24369912	C	6.63100819	1.48174129	-0.18008719
C	-4.90051583	3.13430887	0.01825628	C	-4.84361059	3.17261729	-0.00988190

N	-4.55156636	4.24779190	-0.03192757	N	-4.44695962	4.27437377	-0.05535751
C	-6.74575265	1.65892157	0.18176793	C	-6.73537067	1.77480744	0.11610762
N	-7.91150918	1.63847443	0.26495053	N	-7.90682532	1.79321801	0.17379862
H	-7.18060093	-3.03657508	0.30183372	H	-7.32901150	-2.92564105	0.23390180
H	-5.22524698	-4.58255107	0.21407710	H	-5.43039238	-4.54606877	0.19525709
H	-2.91815987	-3.63373652	0.04437914	H	-3.08481967	-3.67324001	0.07732335
H	-6.85120307	-0.62257070	0.22475984	H	-6.90632270	-0.52342374	0.15904266
H	-2.71193520	2.10272644	-0.10410445	H	-2.68289161	2.05878579	-0.08574134
H	-0.39404091	1.49487748	-0.24021622	H	-0.38706754	1.38548147	-0.18002805
H	0.07616540	-1.54619492	-0.18723103	H	0.05101768	-1.66686079	-0.09367797
H	2.26385202	-2.37792290	-0.27336710	H	2.27825616	-2.46775504	-0.09549151
H	4.66102924	-2.11547008	-0.40179242	H	4.67430102	-2.16117756	-0.19717461
H	4.14051540	2.18517491	-0.57246650	H	4.07166514	2.12757494	-0.53590525
H	1.76425248	1.89911417	-0.41029390	H	1.69869778	1.80048826	-0.39348902
H	6.43742086	-1.77945044	-1.10473067	H	6.39214763	-1.78588669	-1.00658500
H	7.72011857	-0.62860463	-1.42146473	H	7.50799480	-0.56564142	-1.59997302
H	6.80085498	-1.48873667	1.37276569	H	7.28096168	-1.46919021	1.31977556
H	8.29063023	-2.00357193	0.57704767	H	8.58039266	-1.88770509	0.19870315
H	8.65863104	-0.06685199	2.20006465	H	9.18505180	0.06499598	1.73457109
H	9.16080908	0.29292418	0.55200582	H	9.28223124	0.46419332	0.02218421
H	6.55044127	1.19253411	1.88963840	H	6.97682631	1.16182981	1.92402779
H	7.89270396	2.22439801	1.39105787	H	8.09607403	2.29784638	1.16689351
H	6.01470164	2.31347662	-0.25220497	H	5.89704204	2.25808719	0.01714656
H	7.47028849	1.67142628	-0.99604519	H	7.19902355	1.77023858	-1.07430528
